# Immobilization of Cyclooxygenase-2 on Silica Gel Microspheres: Optimization and Characterization

**DOI:** 10.3390/molecules201119670

**Published:** 2015-11-05

**Authors:** Qian Shi, Junhui Chen, Yanlong Wang, Zhaoyong Li, Xianguo Li, Chengjun Sun, Li Zheng

**Affiliations:** 1 College of Chemistry and Chemical Engineering, Ocean University of China, Qingdao 266100, China; srdwjm@sina.com; 2 Research Center for Marine Ecology, The First Institute of Oceanography, State Oceanic Administration, Qingdao 266061, China; wangyanlong92@163.com (Y.W.); lizhaoyong2056@126.com (Z.L.); csun@fio.org.cn (C.S.); zhengli@fio.org.cn (L.Z.)

**Keywords:** cyclooxyenase 2, immobilized enzyme, aminated silica gel, cross linking

## Abstract

In this study, immobilized COX-2 was successfully constructed through glutaraldehyde-mediated covalent coupling on functional silica gel microspheres. The optimum conditions, properties, and morphological characteristics of the immobilized COX-2 were investigated. The optimal immobilization process was as follows: about 0.02 g of aminated silica gel microspheres was activated by 0.25% GA solution for 6 h and mixed with 5 U of free recombinant COX-2 solution. Then, the mixture was shaken for 8 h at 20 °C. Results showed that the immobilized COX-2 produced by this method exhibited excellent biocatalytic activity, equivalent to that of free COX-2 under the test conditions employed. The best biocatalytic activity of immobilized COX-2 appeared at pH 8.0 and still maintained at about 84% (RSD < 7.39%, *n* = 3) at pH 10.0. For temperature tolerance, immobilized COX-2 exhibited its maximum biocatalytic activity at 40 °C and about 68% (RSD < 6.99%, *n* = 3) of the activity was maintained at 60 °C. The immobilized COX-2 retained over 85% (RSD < 7.26%, *n* = 3) of its initial biocatalytic activity after five cycles, and after 10 days storage, the catalytic activity of immobilized COX-2 still maintained at about 95% (RSD < 3.08%, *n* = 3). These characteristics ensured the convenient use of the immobilized COX-2 and reduced its production cost.

## 1. Introduction

Multimetric enzyme–cyclooxygenase (prostaglandin endoperoxide G/H synthase, COX) is a heme-containing enzyme that catalyzes the reaction of arachidonic acid (AA) to produce prostaglandins (PGs) [1]. The PGs present in many tissues can cause inflammation and pain. Undesirable PGs generated from arachidonic acid are mainly catalyzed by COX-2, which is an inducible COX isozyme that plays an important role in inflammation and tumor formation [2,3]. COX-2 also acts on the central nervous to cause hyperalgesia [4] and is related to the occurrence of nephropathy [5,6]. The enzyme has become an important drug target for the discovery and development of anti-inflammatory or anti-tumor drugs because selective inhibition of COX-2 may not only avoid the major side effects associated with COX-1 inhibition but also retain all of the benefits of classical non-steroidal anti-inflammatory drugs [7,8]. The discovery of COX-2-selective inhibitors is an important area of pharmaceutical research [9]. Development of a highly efficient *in vitro* assay method for screening COX-2-selective inhibitors is also a popular research topic.

Over the last several decades, techniques for discovering COX-2-selective inhibitors, including high performance liquid chromatography [10], liquid chromatography-mass spectrometry (LC-MS) [7,8,9], ultrafiltration LC-MS [11,12], virtual screening methods [13,14], bioassays [13,15], and so on, have been increasingly reported. Some of these methods [10,15] used cultured cell lines and were therefore time consuming and required special experimentation skills. The use of commercially available purified or recombinant COX-2 to screen COX-2-selective inhibitors has recently been expanded because assays using purified or recombinant enzymes are simpler, more convenient and less time consuming than cell-based assays. However, commercially available COX-2 is still very expensive, and cost is probably the core bottleneck limiting further applications of the *in vitro* assay method for screening COX-2-selective inhibitors. Using immobilized COX-2 instead of free COX-2 is crucial to overcome the shortcomings (e.g., high cost, non-recyclability, *etc.*) of free enzymes for screening COX-2-selective inhibitors *in vitro*.

Immobilized enzymes present many advantages over free enzymes, including reusability, long-time storage, low cost, and optimum pHs and temperatures that might be shifted to more suitable values, *etc.* [16,17,18]. The stabilization of immobilized enzymes increases when multipoint [17] or mutisubunit [19] immobilization occurs. Furthermore, the selectivity and specificity of immobilized enzymes may vary strongly under different experimental conditions [20]. To achieve these improvements of enzymes’ properties via immobilization, new immobilization system (e.g., new materials, improved applications, visualize technique, *etc.*) and a deeper knowledge of the mechanisms of interaction between enzymes and activated solids both need to be developed [21]. The applications of affinity-immobilization technology, which is based on target-ligand binding interactions, have gradually matured over the years [22,23,24]. Previous successful research cases have encouraged us to develop a novel approach to immobilize COX-2 onto a suitable support. One of the simplest and most gentle coupling methods for enzyme immobilization involves reaction in an aqueous buffer by covalent attachment to water-insoluble carriers via glutaraldehyde (GA) because the buffer provides conditions close to the physiological pH, ionic strength, and temperature [18]. There are two strategies for GA to intermolecularly crosslink enzyme molecules immobilized in supports. The first one is to adsorb the enzyme on the aminated support and then treat the enzyme-support composite with GA to activate all the primary amino groups of supports and the enzyme with just one GA molecule [25]. The second possibility is to use a pre-activated support [26]. With GA preactivated-supports, there are two GA molecules per amino group in the support which gives a quite reactive structure with the amino residues of the enzyme [27]. Different strategies used in experiment may affect the final effect of a chemical modification on the immobilization enzyme properties [28]. To date, only one article has mentioned the immobilization of COX-2 using magnetic particles as a carrier with GA as crosslinker, while the optimization of immobilized process and the characterization of immobilized COX-2 weren’t discussed [7].

In the present study, functionalized porous SiO_2_ microspheres were prepared and COX-2 was immobilized on the surface of these microspheres with GA as crosslinker. Immobilization conditions, such as GA dosage, reaction pH, reaction temperature and enzyme dosage were optimized, according to the relative activity of the immobilized COX-2, and the properties of the immobilized COX-2 were investigated.

## 2. Results and Discussion

### 2.1. Optimization of the Immobilization Conditions

**Scheme 1:**
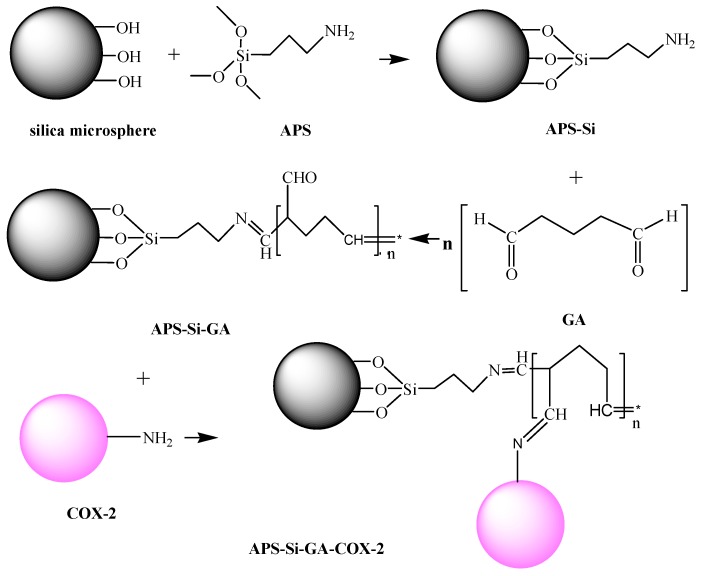
Schematic of the synthesis of NH_2_-modified SiO_2_ particles and construction of the cross-linked immobilizedCOX-2 via GA chemistry (Schiff bases model).

Silica particles are hydrophilic, biocompatible, and stable in most biosystems [24]. Moreover, silica chemistry is fairly well known, and standard chemical protocols can be followed to conjugate various biomolecules to the silica surface [18,29], which is an excellent solid support for enzyme immobilization [30,31]. NH_2_-modified SiO_2_ particles were chosen for the immobilization experiment in this study, and the immobilization conditions, including the dosage of the crosslinking agent, reaction pH, reaction temperature, and enzyme dosage were optimized. The procedures for the preparation of NH_2_-modified SiO_2_ particles and the immobilization of COX-2 are illustrated in Scheme 1. From the scheme, we can see that GA doesn’t react with enzyme inits free form, but as an unsaturated polymer, which gives imino bonds stabilized by conjugation [32]. The structure of the GA relevant for the modification of enzymes and supports is not a linear one, but some kind of fairly stable ring [33].

#### 2.1.1. Effect of GA Concentration

**Figure 1:**
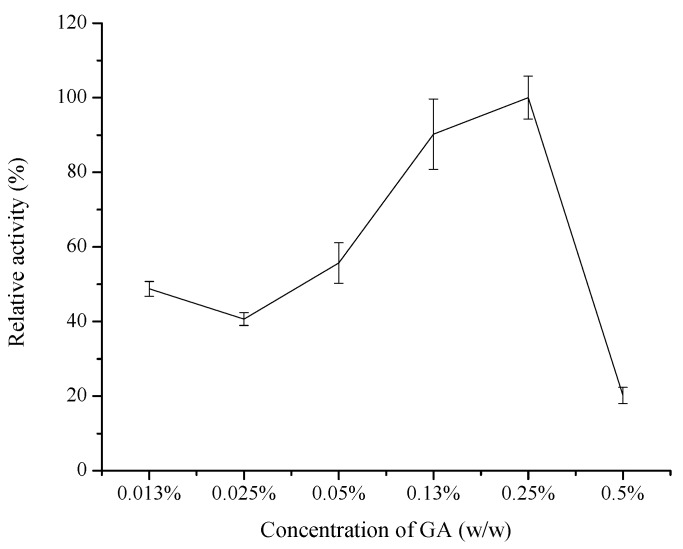
Effect of GA concentrations on the relative activity of the immobilized COX-2 (*n* = 3).

GA, a linear, 5-carbon dialdehyde is a pungent oily liquid which can be solubilized in water, as well as in organic solvents [34]. It reacts with amine groups at around neutral pH [35] and is more efficient than other aldehydes in generating thermally and chemically stable crosslinks [36]. The GA concentrations must be carefully investigated in order to obtain water-insoluble enzyme derivatives via crosslinking because low concentrations of enzyme and GA tend to induce intramolecular crosslinking by enhancing the probability that GA functional groups will react with the same enzyme molecule [18]. Thus, the effects of different final concentrations (*i.e.*, 0.013%, 0.025%, 0.05%, 0.13%, 0.25%, and 0.5%, *w*/*w*) of GA in the buffer were evaluated. The effect of GA dosage during immobilization process on the activity of the immobilized COX-2 is shown in Figure 1. The relative activity of immobilized COX-2 increased as the concentration of GA increased in the range of 0.013%–0.25% and then decreased as the GA concentration exceeded 0.25%. The results demonstrated that low GA concentrations are unable to provide sufficient aldehyde groups (crosslinkages) to react with the enzyme. By contrast, excess GA may produce steric hindrance and decrease the accessibility and accommodation of the substrate to the enzyme, which would lead to decreases in the relative activity of the immobilized enzyme [18,34]. Thus, the GA concentration was optimized to 0.25% in succeeding experiments. The relationship between GA concentration and relative activity was different from the literature [27]. This may be contribute to the high versatility that GA expressed in the process of immobilization, which greatly affect the final effect of a chemical modification on the immobilization enzyme properties [28].

#### 2.1.2. Effect of Reaction Solution pH

**Figure 2:**
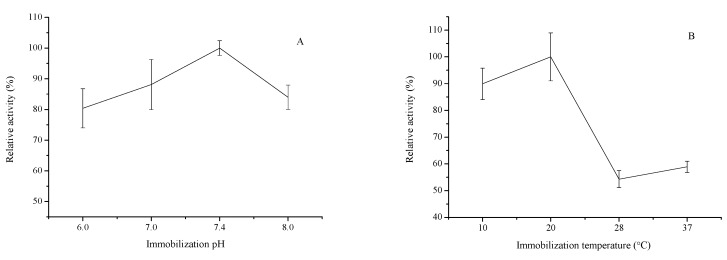

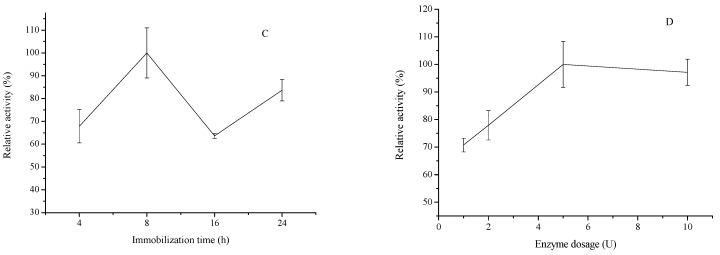
Effect of immobilization pH (**A**); reaction temperature (**B**); immobilization time (**C**) and enzyme dosage (**D**) on the relative activity of the immobilized COX-2 (*n* = 3).

The pH value is one of the key parameters that may turn subunit dissociation into the first step of the inactivation of multimeric enzymes [19]. During immobilization, improper solution pH can change the three-dimensional shape and activity of the enzymes [37], resulting in decreases in the relative activity of the immobilized enzyme. Hence, the effect of solution pH on the immobilized COX-2 was investigated by incubating the preparations in buffers of varying pH ranging from 6.0 to 8.0, as depicted in Figure 2A. The immobilized COX-2 exhibited maximum activity at pH 7.4. When the solution pH was less than 7.4, the immobilized COX-2 activity increased with increasing solution pH but decreased when pH > 7.4 because of enzyme inactivation [18]. Therefore, pH 7.4 was selected as the best reaction solution pH for subsequent immobilization experiments.

#### 2.1.3. Effect of Reaction Temperature and Time

Different immobilization temperatures affect the enzymatic reaction rate and protease denaturation. To examine the effect of reaction temperature on the relative activity of the immobilized COX-2, the reaction was performed at temperatures ranging from 10 °C to 37 °C (Figure 2B). When the temperature was lower than 20 °C, the activity of the immobilized enzyme increased with increasing temperature, yielding an activity maximum at 20 °C. As the temperature further increased, the relative activity of the immobilized enzyme decreased because of enzyme inactivation at high temperatures.

The effects of reaction time on the relative activity of the immobilized COX-2 were also studied. The results shown in Figure 2C demonstrate that the relative activity of immobilized COX-2 is highest after 8 h of immobilization time. Consequently, the reaction temperature and time were optimized to 20 °C and 8 h, respectively, in succeeding experiments.

#### 2.1.4. Effect of Enzyme Dosage

The dosage of free enzyme is an important parameter affecting enzyme immobilization. For multimeric enzymes, dissociation of enzyme subunits displays a direct dependence between enzyme concentration and enzyme stability [38,39]: enzyme stability increases with increasing of enzyme concentrations. However, with increasing free enzyme concentration, steric hindrance and non-specific adsorption also increase. Thus, the effect of free enzyme dosage on the relative activity of the immobilized COX-2 was investigated. As shown in Figure 2D, relative activity of immobilized COX-2 was only 70% (RSD < 2.49%, *n* = 3) when the dosage of COX-2 was 1 U. The immobilization proceeds better with increasing free COX-2 dosage, and the free COX-2 dosage ensuring optimum activity of immobilized COX-2 was be 5 U. Thus, the dosage of the free enzyme was optimized to 5 U in subsequent experiment.

In summary, the optimum immobilization condition was as follows: about 0.02 g of aminated silica gel microspheres was activated by 0.25% GA solution for 6 h and mixed with 5 U of free recombinant COX-2 solution after redundant GA removed. Then, the mixture was shaken for 8 h at 20 °C. The immobilized COX-2 was separately washed with 3.5% (*w*/*v*) NaCl solution, ultrapure water, and PBS thrice and then stored in 1 mL PBS at 4 °C. Then free COX-2 was selected as control, and biocatalytic activity of the immobilized COX-2 obtained under the optimum immobilization condition was compared with that of free COX-2. Biocatalytic activity of the immobilized COX-2 was equivalent to that of free COX-2. The increase of activity may be due to the rigidification or un-aggregation of immobilized enzyme [40].

### 2.2. Characterization

**Figure 3:**
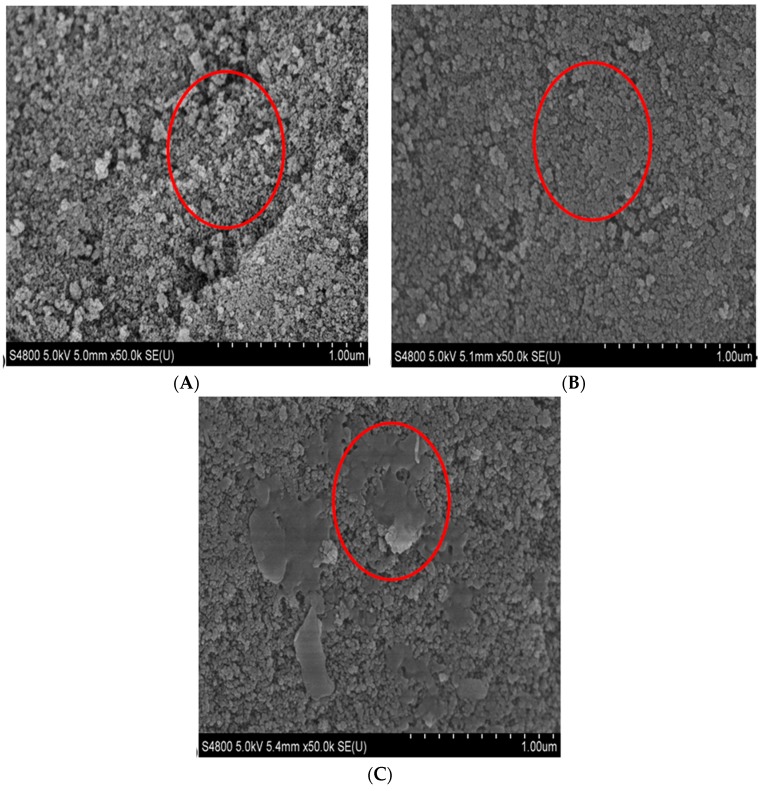
FESEM images of (**A**) the activated silica gel; (**B**) APS-Si; and (**C**) APS-Si-GA-COX-2.

Field emission scanning electron microscopy (FESEM) images of untreated and NH_2_-modified SiO_2_ particles before and after enzyme immobilization are displayed in Figure 3. The activated silica gel surface was uneven (Figure 3A), and the surface of APS-Si tended to be flat (Figure 3B). Figure 3C shows that some sheet-like materials appear on the surface of APS-Si-COX-2, thereby demonstrating that the free enzyme visibly bound to the carriers.

### 2.3. Properties of Immobilized Cyclooxyenase-2

#### 2.3.1. Effect of Oscillation, pH and Temperature on the Biocatalytic Activity of Immobilized COX-2

**Figure 4:**
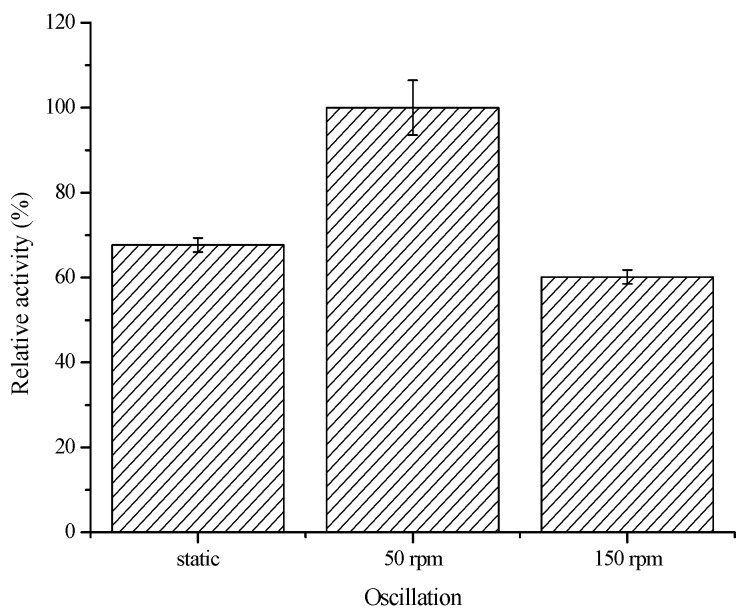
Effect of oscillation on the biocatalytic activity of the immobilized COX-2 (*n* = 3).

The biocatalytic activity of the immobilized enzyme is greatly dependent on the pH, temperature, and status of the reaction solution. First, the effect of oscillation on the biocatalytic activity of the immobilized COX-2 was investigated. The reaction solution was placed understatic, gently shaken (50 rpm), or vigorously shaken (150 rpm) conditions at 37 °C for 10 min. As shown in Figure 4, gentle oscillation favored the biocatalytic reaction. Under the static and vigorous shaking conditions, the biocatalytic activities of the immobilized COX-2 were about 60%–70% of the activity observed in the gently shaken system.

**Figure 5:**
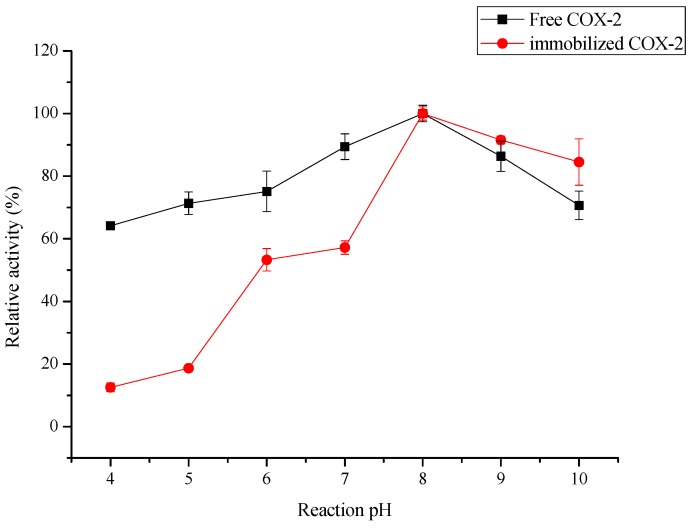
Effect of reaction pH on the biocatalytic activity of the immobilized COX-2 and free COX-2 (*n* = 3).

Changes in pH can affect the enzyme conformation and degree of dissociation of the substrate, thereby affecting the binding and biocatalytic effects between enzyme molecules and the substrate [41]. In this study, the effects of reaction solution pH in the range of 4.0–10.0 on the biocatalytic activity of the free and immobilized COX-2 were studied, and relevant results are shown in Figure 5.

The immobilized COX-2 showed maximum biocatalytic activity at pH 8.0. Much lower or higher pH values led to decreases in the biocatalytic activity of the immobilized COX-2. Hereinto, acidic conditions affected the biocatalytic activity of immobilized COX-2 more than alkaline conditions. This is because of the Schiff bases formed under acidic and neutral conditions are unstable, regenerating both the aldehyde and the amino groups [33], but the fact that the biocatalytic activities of immobilized COX-2 still remain about 80% at pH 10.0 demonstrates that the immobilized COX-2 had good tolerance for higher pH values. The relative activity of free COX-2 increased gently, almost the same as the immobilized COX-2. The immobilization process changed the pH tolerance of the enzyme; the biocatalytic activity of the immobilized COX-2 at pH 4 was 50% lower than that of free COX-2.

**Figure 6:**
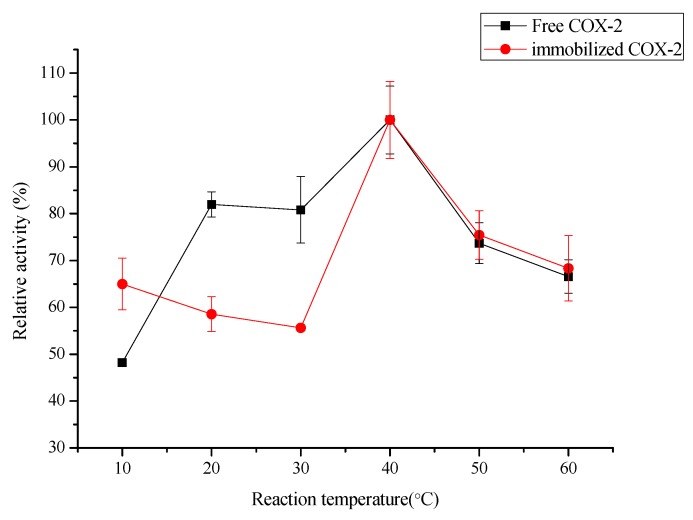
Effect of temperature on the biocatalytic activity of the immobilized COX-2 and free COX-2 (*n* = 3).

The temperature of the reaction solution is another important parameter affecting the biocatalytic activity of the immobilized enzyme. To examine the effect of temperature on its biocatalytic activity, the free and immobilized COX-2 was incubated in different temperatures (*i.e.*, 10, 20, 30, 40, 50, and 60 °C) for 10 min, after which its biocatalytic activity was measured. As shown in Figure 6, the relative activity tendency of the free and immobilized COX-2 was almost the same. The optimum reaction temperature for both enzymes was 40 °C. The reduction in the biocatalytic activity of free and immobilized COX-2 at lower or higher temperatures is associated with damage to the quaternary structure in the accessible surface area under extreme heating or cooling [42]. The free COX-2 was unable to maintain the biocatalytic activity at low temperature, only about 58% of the immobilized COX-2 at 10 °C. The reason for the inactivation of free COX-2 is the dissociation of the enzyme subunits or the dissociation of the enzyme subunits or the loss of their correctly assembled structures [38,39]. In general, the immobilized COX-2 had a better relative activity than the free COX-2. The immobilized COX-2 exhibited around 60% of its maximum biocatalytic activity at temperatures ranging from 10 °C to 30 °C. At 60 °C, 70% activity was still maintained by the immobilized COX-2. The activity of immobilized COX-2 under high temperature was better than that of free COX-2 was due to the immobilization-induced rigidification, which avoided distortion of the enzyme structure [40].

#### 2.3.2. Reusability and Storage Stability

**Figure 7:**
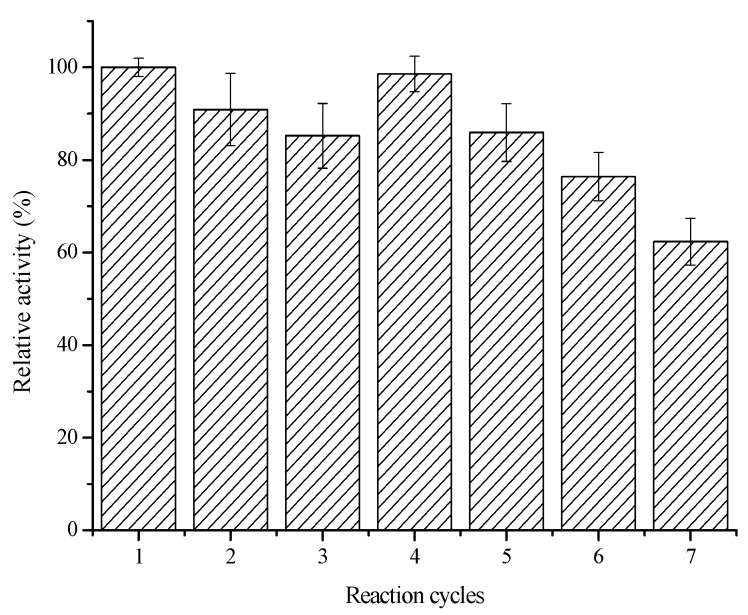
Reusability of the immobilized COX-2 (*n* = 3).

To expand the practical applications of immobilized enzymes, reusability is an important consideration [43]. To test its reusability, the immobilized COX-2 was reused seven times and its biocatalytic activity was recorded after each recovery. Figure 7 shows the relative biocatalytic activities of the immobilized COX-2 obtained after recycling; here, the initial biocatalytic activity is defined as 100%. The immobilized COX-2 retained over 85% (RSD < 7.26%, *n* = 3) of its initial biocatalytic activity after five cycles and 60% (RSD < 8.12%, *n* = 3) of its initial biocatalytic activity after seven cycles, which indicates that it has appropriate stability and can be reused.

**Figure 8:**
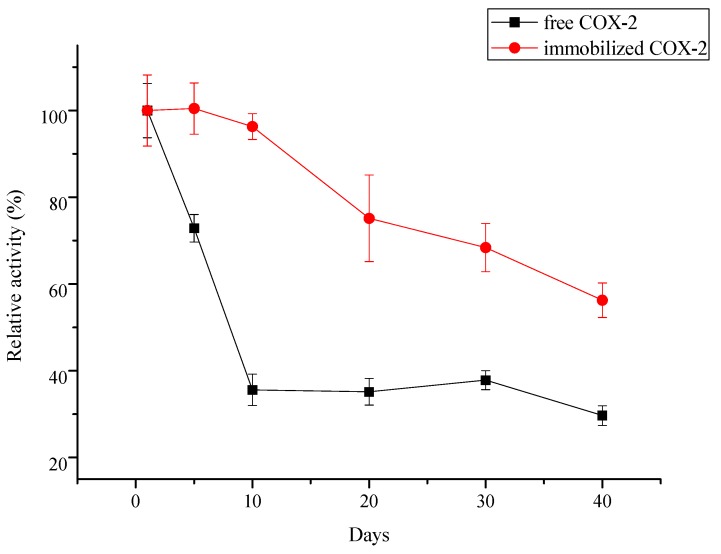
Storage stability of the immobilized COX-2 (*n* = 3).

The storage stability of the immobilized COX-2 was further investigated over the course of 40 days. The free COX-2 was stored in storage solution and the immobilized COX-2 was stored in 0.1 mol/L PBS solution at a temperature of 4 °C. The results are presented in Figure 8. About 66% activity of free COX-2 was lost during the first 10 days and the relative activity maintained in that value about 20 days. For immobilized COX-2, no significant loss of biocatalytic activity was observed after 5 days of storage, and over 95% (RSD < 3.08%, *n* = 3) biocatalytic activity relative to its initial activity was observed on day 10; such activity is even better than that described in the literature [7]. The increased enzyme stability is attributed to enzyme rigidfication. COX-2 is a multimeric enzyme [44], and immobilization of COX-2 may involve multiple subunits, which prevents the enzyme dissociation [19]. With increasing storage time, the activity of the immobilized COX-2 gradually decreased. After 40 days of storage, the activity of the immobilized COX-2 dropped to below 60% of its initial activity. Overall, the immobilized COX-2 exhibited good storage stability, thereby ensuring its convenient and reduced production cost.

## 3. Experimental Section

### 3.1. Materials and Reagents

SiO_2_ (grade 643, 200–425 mesh, 150 Å, 99%, Davisil), human recombinant COX-2, arachidonic acid (AA), prostaglandin E_2_ (PGE_2_), and sodium diethyldithiocarbamate were purchased from Sigma (St. Louis, MO, USA). Tris (hydroxymethyl) aminomethane was purchased from J & K Chemical (Beijing, China). HPLC-grade acetonitrile and methanol were purchased from ACS Company Inc. (Wilmingtong, DE, USA). γ-Aminopropyltrimethoxysilane (APS) was purchased from Alfa Aesar (Heysam, Lancs, UK). Purified water (18.2 ΩM) was prepared by using a Milli-Q water purification system (Millipore, Bedford, MA, USA). Phosphate buffer solution (100 mmol/L, pH 7.4) was selected and all other reagents used were of GR grade.

### 3.2. Preparation of NH2-Modified Silica Microspheres

Exactly 2.00 g of silica microspheres was boiled with 5% HCl for 45 min to remove absorbed organic compounds on the surface of the particles. Rinsing with ultrapure water followed until the solution was of neutral pH. After rinsing, the silica microspheres were dried at 80 °C for 24 h. Aminated surfaces were manufactured according to our previous study [45]. Briefly, 0.50 g of activated silica microspheres was treated with 20 mL of 5% APS–toluene solution at 90 °C for 24 h. The modified silica (APS-Si) was then rinsed with acetone to remove the physically absorbed silane coupling agent from the surface. After acetone volatilization, the APS-Si was dried at 110 °C for 6 h.

### 3.3. Immobilization of Cyclooxyenase-2

COX-2 storage solution was prepared with 80 mM Tris-HCl, pH 8.0 with 0.1% TWEEN 20 and 300 μmol/L sodium diethyldithiocarbamate and 10% glycerol. Free COX-2 was dissolved in storage solution to a final concentration of 100 U/mL. The solid support (0.02 g) was dispersed in 2.0 mL of PBS for full swelling, after which 10 μL of 50% (*w*/*w*) GA was added to give a final concentration of 0.25% (*w*/*w*). The APS-Si was reacted with GA at 28 °C for 6 h and then washed with an excess of ultrapure water to remove the redundant GA on the surface. The immobilization procedure was carried out by adding 1 mL of PBS and 50 μL of COX-2 (5 U) to the GA-treated silica carrier at 20 °C in a rocking incubator for 8 h. The immobilized COX-2 was separately washed with 3.5% (*w*/*v*) NaCl solution, ultrapure water, and PBS thrice to remove non-specifically bound COX-2 and stored in 1 mL PBS at 4 °C for further experimentation. Three replications of all assays were conducted in our study.

### 3.4. Characterization

A sputter coater (E-1045, Hitachi, Japan) was used to fabricate Au thin films. FESEM (S4800, Hitachi, Japan) was used to observe the morphology of the microspheres. A small amount of the samples (activated silica microspheres, APS-Si powder, and carrier bonded with the enzyme) was adhered to the sample holder and then coated with Au using the sputter coater. The treated silica samples were transferred to the FESEM sample pool to determine their surface morphological characteristics.

### 3.5. Determination of Immobilized Cyclooxyenase-2 Activity

The biocatalytic activity of immobilized COX-2 was determined according to the literature [9] with slight modifications. Briefly, in an Eppendorf tube, 4 μL AA (500 mg/L) was added to 200 μL of the immobilized enzyme, and the reaction was terminated at 37 °C after 10 min. Afterward, the generated PGE_2_ was extracted using 1500 μL of hexane/ethyl acetate (50:50, *v*/*v*), and the extraction process was repeated another two times. The organic phase was removed, dried with nitrogen, and reconstituted in 100 μL of methanol for analysis by using LC-MS/MS. The activity of immobilized enzyme based on the production of PGE_2_ under optimal condition was defined as 100%, and relative activity was defined as the ratio of the yield of PGE_2_ in other conditions to that of optimum condition.

Chromatographic separation was performed on an analytical Agilent Poroshell120 C18 column (2.7 μm × 3.0 mm × 100 mm; Agilent, Santa Clara, CA, USA), and the eluent was delivered by an Agilent 1200 HPLC system. The mobile phase for PGE_2_separation consisted of solvent A (water) and solvent B (acetonitrile); 5 min of isocratic elution of 0–5 min with 35% B was performed. The mobile phase flow rate was 0.4 mL/min at room temperature and the injection volume was 10 μL.

A 6320 Series ion-trap mass spectrometer (Agilent Technologies, Wilmington, DE, USA) equipped with an ESI interface in negative mode was used to detect PGE_2_. The instrument was operated in full-scan and multiple reaction monitoring (MRM) modes. Other instrument parameters were as follows: scan range, 100–500 *m*/*z*; nebulizer pressure (N_2_), 35 psi; drying gas (N_2_), 11 L/min; and drying gas temperature, 325 °C. The deprotonated molecules of [M − H]^−^ (*m*/*z* 351) corresponding to PGE_2_ were selected for collision-induced dissociation at a collision energy of 1.0 V. During MRM, three fragment ions of *m*/*z* 333, 315, and 271 were acquired; these ions correspond to [M − H − H_2_O]^−^, [M − H − 2H_2_O]^−^, and [M − H − 2H_2_O − CO_2_]^−^, respectively. PGE_2_ was measured by recording the signal for the deprotonated molecules of *m*/*z* 351 to fragment ion of *m*/*z* 333.

### 3.6. Reusability of Immobilized Enzyme

After each assay, the immobilized COX-2 was taken out from the reaction medium by centrifugation and was washed separately with 3.5% NaCl, ultra-pure water, and phosphate buffered saline (PBS, 0.1 mol/L, pH 7.4) thrice to remove the substrate and adhered products. The immobilized enzyme recovered by this procedure was used repeatedly. The activity determined for the first time was considered as control (100%) for the calculation of remaining percentage activity after each use.

## 4. Conclusions

In this study, functionalized SiO_2_ microspheres were prepared and COX-2 was successfully immobilized on the surface of NH_2_-modified SiO_2_ particles by using GA as the crosslinking agent. The proposed immobilized COX-2 exhibited good reusability and storage stability. The activity of the immobilized COX-2 exceeded 60% of its initial biocatalytic activity after seven cycles of reuse. These results demonstrate the efficient use and low cost of the immobilized COX-2. The immobilized COX-2 proposed in this work may be used as an enzyme catalyst for the enzymatic transformation of AA and facilitate the development of other *in vitro* assay methods for screening of COX-2-selective inhibitors.
